# Open-ocean polynyas and deep convection in the Southern Ocean

**DOI:** 10.1038/s41598-019-43466-2

**Published:** 2019-05-06

**Authors:** Woo Geun Cheon, Arnold L. Gordon

**Affiliations:** 1 Maritime Technology Research Institute, Agency for Defense Development, Changwon, Republic of Korea; 2 Lamont-Doherty Earth Observatory of Columbia University, Palisades, NY USA

**Keywords:** Physical oceanography, Physical oceanography, Cryospheric science

## Abstract

An open-ocean polynya is a large ice-free area surrounded by sea ice. The Maud Rise Polynya in the Southern Ocean occasionally occurs during the austral winter and spring seasons in the vicinity of Maud Rise near the Greenwich Meridian. In the mid-1970s the Maud Rise Polynya served as a precursor to the more persistent, larger Weddell Polynya associated with intensive open-ocean deep convection. However, the Maud Rise Polynya generally does not lead to a Weddell Polynya, as was the situation in the September to November of 2017 occurrence of a strong Maud Rise Polynya. Using diverse, long-term observation and reanalysis data, we found that a combination of weakly stratified ocean near Maud Rise and a wind induced spin-up of the cyclonic Weddell Gyre played a crucial role in generating the 2017 Maud Rise Polynya. More specifically, the enhanced flow over the southwestern flank of Maud Rise intensified eddy activity, weakening and raising the pycnocline. However, in 2018 the formation of a Weddell Polynya was hindered by relatively low surface salinity associated with the positive Southern Annular Mode, in contrast to the 1970s’ condition of a prolonged, negative Southern Annular Mode that induced a saltier surface layer and weaker pycnocline.

## Introduction

In the Southern Ocean there are two modes in which surface water can attain sufficient density to descend into the deep ocean^1^. The more common mode stems from the dense shelf water formed within coastal polynyas mainly in the Weddell and Ross Seas^2,3^, which then descends over the continental slope as gravity currents or plumes^4^. The continental margin mode, also known as near-boundary convection^3^, in the present-day climate system is the dominant contributor to the formation of Antarctic Bottom Water that spreads across the global ocean. Open-ocean deep convection, which is closely linked to open-ocean polynyas^3,5–8^, is another mode for the Southern Ocean surface water masses to sink into the deep ocean.

**Figure 1:**
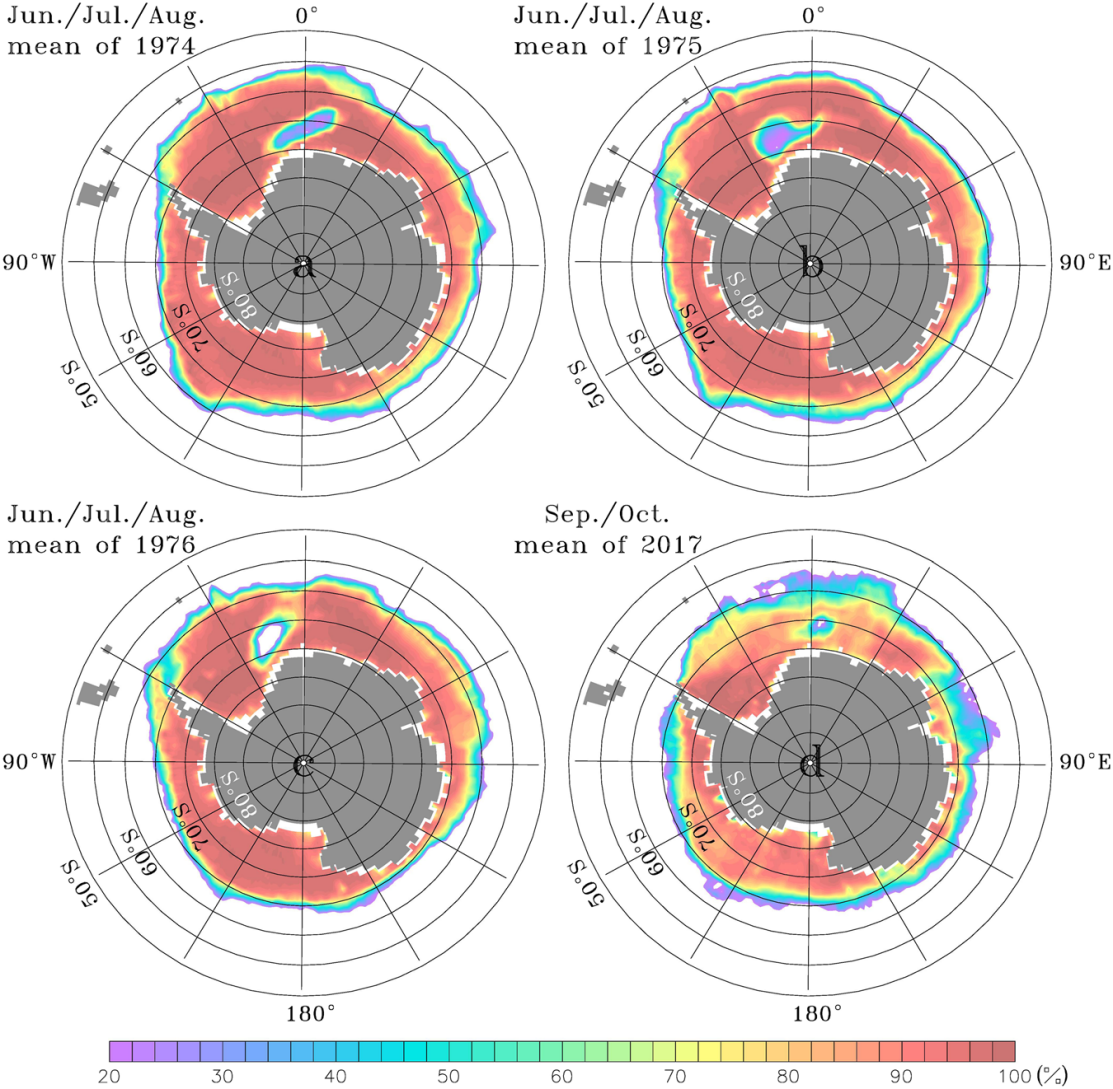
(**a**–**c**) June/July/August mean sea-ice concentrations of 1974/1975/1976 and (**d**) September/October mean sea-ice concentration of 2017 derived from the HadISST data. Areas of low sea ice concentration (blue/purple/white colors) enclosed within areas of high sea ice concentration (orange/red colors) between 30°W and 10°E indicate the Weddell Polynya occurring at 1970s and the Maud Rise Polynya occurring at 2017, respectively.

**Figure 2:**
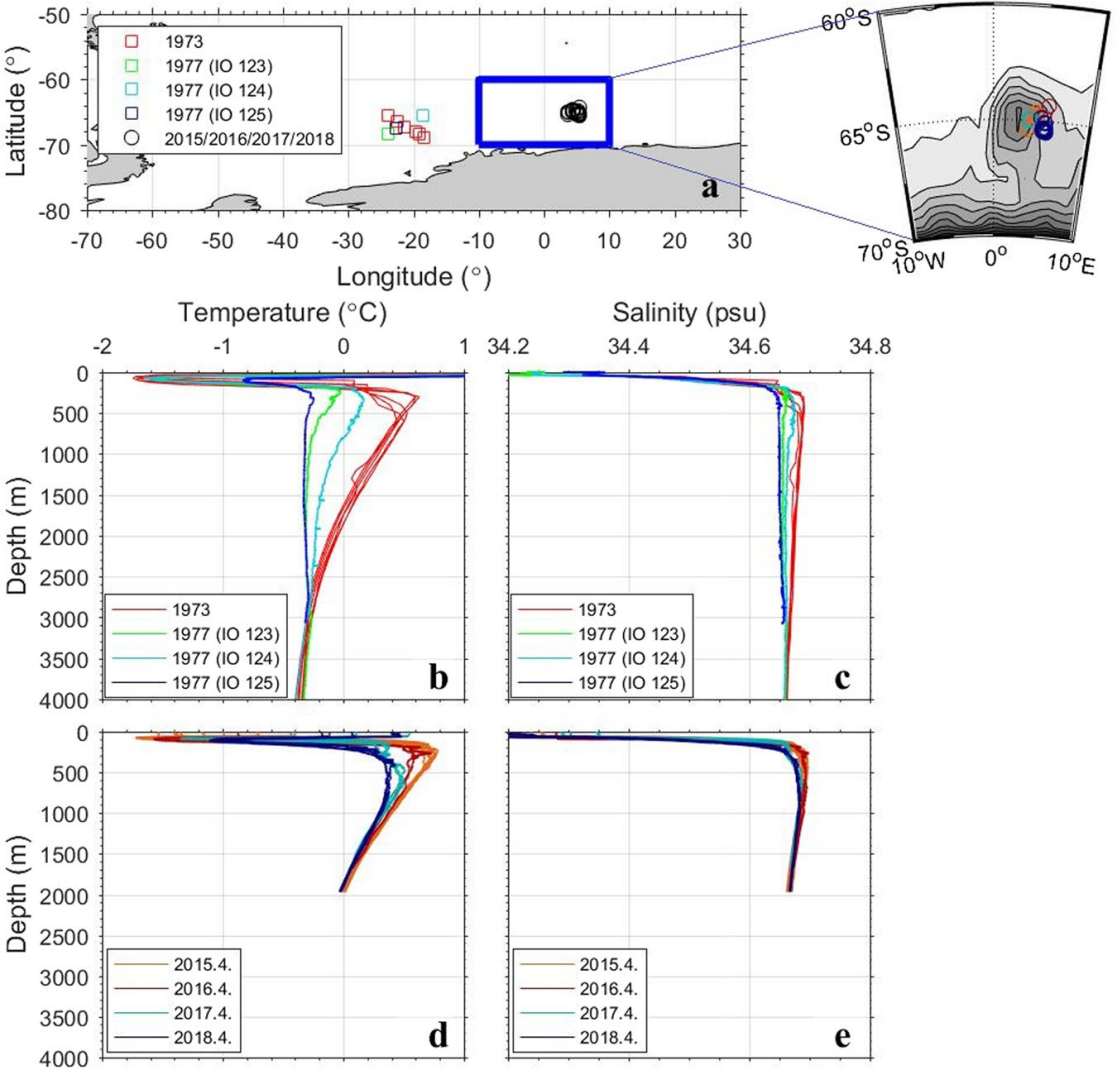
(**a**) Positions of hydrographic stations to measure water properties by use of CTD (1977, Conductivity-Temperature-Depth recorder), STD (1973, Salinity-Temperature-Depth recorder), and Argo floats (2015/2016/2017/2018). Vertical profiles of (**b**,**d**) temperature and (**c**,**e**) salinity measured before and after (**b**,**c**) the 1970s’ Weddell Polynya and (**d**,**e**) the 2017 Maud Rise Polynya.

Since the first satellite observation of the Antarctic winter sea ice cover was available in 1972, a persistent, larger-scale open-ocean polynya was only once observed in the Weddell Sea. Known as the Weddell Polynya (WP)^9^, this polynya remained open for three consecutive winters from 1974 to 1976 and had an average size of about 250 × 10^3^ km^2^ (Fig. 1a–c). As shown in Fig. 2b,c, the Weddell Deep Water (WDW) was significantly cooled and freshened to nearly 2700-m depth by open-ocean deep convection^5,6^. During three years of the WP, the total heat loss of the WDW was estimated to be 12.6 × 10^20^ Joules^6^.

**Figure 3:**
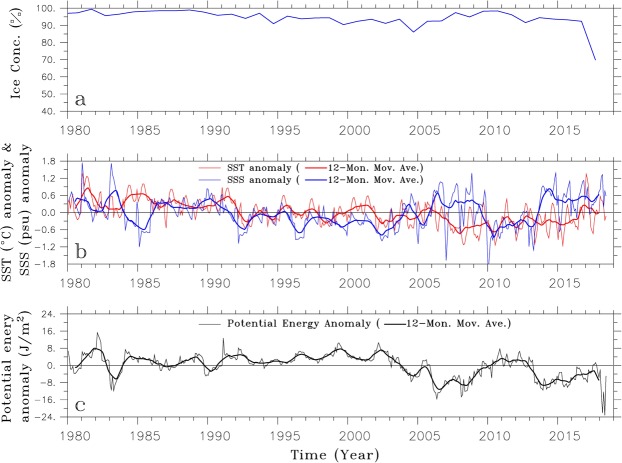
Time series of (**a**) sea ice concentration, (**b**) (red) SST and (blue) SSS anomalies, and (**c**) potential energy anomalies averaged over the region (0°–10°E/67°S–62°S) in which the Maud Rise polynyas have occurred. Thicker lines denote data filtered by the 12-month moving average.

Smaller, less persistent open-ocean polynyas are also observed in the vicinity of Maud Rise located in the Weddell Sea during late winter or early spring^10^. A Maud Rise Polynya (MRP) occurred in October-November of 1973, just one year prior to the occurrence of the WP in the mid-1970s, and was thus inferred to be its precursor^11^. The occurrence of the WP and the MRP led to a decrease in the heat content of WDW in the vicinity where they occurred^6,12^. Recently, following the MRP development in 2016^13^, a longer-lived, larger MRP was observed from September to November of 2017^14^. Its sea ice concentration declined to below 10% (nearly ice-free), and its size extended over ~50 × 10^3^ km^2^ (Fig. 1d). The 2017 MRP (MRP2017, hereafter) is the largest event since 1980 (Fig. 3a). However, the MRP2017 did not generate a WP in austral winter of 2018, as might have been expected, nor was there a repeat of the MRP in 2018.

Within the Southern Ocean, south of the Antarctic Circumpolar Current the thermohaline stratification is characterized by cold, low salinity surface water separated by a rather weak pycnocline from relatively warm, saline deep water. The pycnocline is shallowed within the larger cyclonic circulation feature in the Weddell Sea, referred to as the Weddell Gyre. Measurements of the thermohaline stratification within the Weddell Gyre before and after the WP reveal active winter open-ocean deep convection, cooling the WDW and by injecting warm water into the surface layer, acting to prevent overlying sea ice cover, inducing the WP^4^.

For this very unusual, natural phenomenon, numerous studies have been performed in order to find the occurrence mechanism of WP event. However, since the 1970s’ WP and the previous MRPs except the MRP2017 occurred during the time when the observation on the Weddell Sea were still not active, these studies were based only on numerical models from simple models^15–17^ to fully coupled climate models^18–25^ and very limited, short-term observation data measured in the Weddell Sea^26–29^, in which the models did not include a data assimilation process. In this paper, we investigate the conditions that govern the generation of the MRP2017 by use of diverse, long-term observation and reanalysis data and prove the hitherto hypotheses proposed by the previous studies. The Sea Surface Height (SSH) data provided by AVISO and high-resolution (1/12° × 1/12° in horizontal direction) reanalysis data from the Hybrid Coordinate Ocean Model (HYCOM) of the Naval Research Laboratory are in this study used to estimate variation of the Weddell Gyre intensity since the mid-1990s, along with *in situ* observational data of the thermohaline stratification. We focus on 4 questions: 1- how has been the thermohaline stratification of the upper ocean weakened within the Weddell Gyre before the occurrence of MRP2017? 2- how did the basin-scale atmosphere circulation over the Weddell Sea intensify the underlying cyclonic Weddell Gyre circulation? 3- what events occurred in the vicinity of Maud Rise and led to the MRP2017? and 4- why didn’t the MRP2017 lead to a larger-scale, more persistent WP event, as materialized in the 1970s?

## Results

To compare ocean stratification conditions of the 1970s’ WP with those of the MRP2017, vertical profiles of temperature and salinity measured during the respective periods are inspected (Fig. 2). A series of STD (Salinity-Temperature-Depth recorder) hydrographic station data were obtained by Glacier in January-February of 1973, showing the oceanic state before the occurrence of the 1970s’ WP, and the CTD (Conductivity-Temperature-Depth recorder) hydrographic station data were obtained by *Islas Orcadas* (IO) in February of 1977^5,6^, depicting the ocean stratification after the WP. The Argo profile data included in the EN4.2.1^30^ dataset of the Met Office Hadley Centre was employed to show changes in temperature and salinity in the area of MRP2017 (3.5°–5.5°E/65.5°S–64°S) from 2015 to 2018. The Argo data from April of 2015 and April of 2016 reveal the oceanic state in the austral fall before the occurrence of the MRP, and those measured in April of 2017 and April of 2018 depict the oceanic state after the MRP.

During the WP (1974–1976), open-ocean convection extended to nearly 2700 m, cooling and freshening the WDW (Fig. 2b,c)^5,6^. The water column measured at the IO 125 station shows very unique, deep water formation of chimney type^3^. The chimney formation is a key signature of open-ocean deep convection, revealing a water column that is homogeneous from the surface to the base of the convection layer. Since there were no observations in the area in 1974, 1975, and 1976, the deep reaching convection might occur within the time frame of less than three years. In the WP simulated in a sea-ice – ocean coupled general circulation model^31^, it took only one year for the surface water masses to sink to the sea floor.

The stratification in the Maud Rise vicinity displays some similarities to the stratification change associated with the WP (Fig. 2b–e). Between the austral fall (April) of 2015 and that of 2018 there was a significant cooling to a depth of 1000 m, along with a slight freshening, indicative of mixing between the surface layer and the WDW. After the longer-lived, larger-scale MRP occurred from September to November of 2017, the water from pycnocline depth to about 1000-m depth was completely mixed. While the occurrence of MRP2017 and its impact on the underlying ocean were very similar with the situation of 1970s’ WP, the MRP2017 did not lead to a WP. Now we elucidate the occurrence mechanism of open-ocean polynya in the Southern Ocean by analyzing the MRP2017 in depth.

### Destabilization of the upper ocean

The sea ice concentration derived from the HadISST data set^32^ (averaged over 0°–10°E/67°S–62°S for September and October), reveals where and when the MRP occurred (Fig. 3a). As previously stated, the MRP2017 is the largest MRP since 1980. To inspect the variation of surface ocean in the vicinity of Maud Rise, anomalies are calculated from the monthly-mean sea surface temperature (SST) and salinity (SSS) averaged over 0°–10°E/67°S–62°S (Fig. 3b). The SST and SSS are derived from the objective analyses form of the EN4.2.1 dataset, which includes the World Ocean Database 2009 (WOD2009) and the Global Temperature and Salinity Profile Program (GTSPP) data from 1990 onward. Since 1980, there are three multi-year periods of positive SSS anomalies: 1986 to 1991, 2006 to 2009 and 2013 to the present. The latter positive anomaly is the largest. The SST anomaly gradually decreases since 1980 and maintains its negative state from 2006 to the present. Therefore, dense surface water occurs from 2006 to 2009 and from 2013 to the present, weakening the ocean stratification near the Maud Rise and setting up preconditions for the MRP.

The stratification is assessed by calculating the available potential energy anomaly^33^ defined as the amount of mechanical energy needed for the water column to be entirely mixed with a given density stratification (Eq. 1):

1 $${\rm{APE}}=\frac{1}{H}{\int }_{-H}^{0}(\bar{\rho }-\rho ){gzdz}{.}$$

The water depth (*H*) is taken to be 1000 m, and the gravitational acceleration (*g*) is 9.8 m/s^2^ and $\bar{\rho }$ is the horizontally averaged density. A large potential energy anomaly denotes a relatively stable oceanic stratified state, while a small anomaly denotes a relatively weakly-stratified state. In agreement with a combination of the SSS and SST anomalies, there are two periods showing clearly negative potential energy anomalies persisting for several years: 2006 to 2009 and 2013 to the present (Fig. 3c), which implies that the ocean in the vicinity of Maud Rise is weakly-stratified and thus enhances the likelihood of the cold surface water mixing with the warmer deep water, contributing to melting the overlying sea ice and creating the MRP. Consistent with the water column measured in April of 2018 (Fig. 2d,e), the potential energy anomaly drastically decreases after the occurrence of MRP2017, denoting weaker stratification between the surface and deep layers.

### The atmosphere-to ocean dynamic interaction

**Figure 4:**
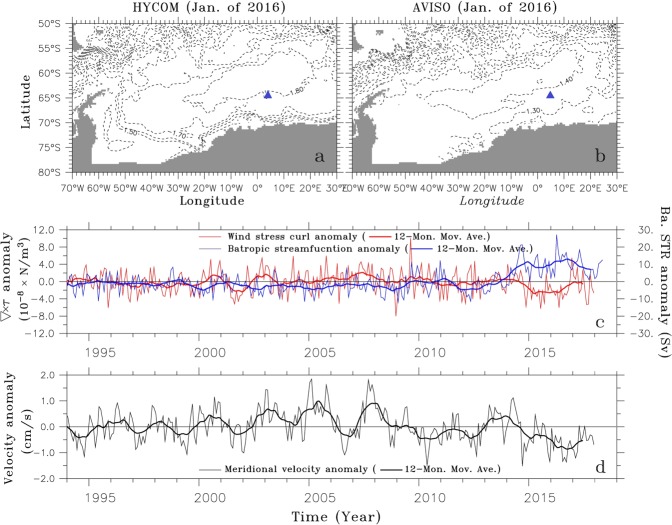
Horizontal distributions of sea surface height in January of 2016 derived from (**a**) AVISO and (**b**) HYCOM and time series of (**c**) meridional velocity anomaly derived from AVISO’s absolute dynamic topography and (**d**) (red) minimum wind stress curl derived from ECMWF ERA-Interim and (blue lines) maximum barotropic streamfunction anomalies derived from HYCOM. The meridional velocity anomaly is averaged over 30°E–40°E/60°S–50°S, where the eastern limb of Weddell gyre is strongest, and where the sea ice does not cover, and the wind stress curl and barotropic streamfunction are averaged over 60 W°–20°E/80°S–60°S covering the Weddell Sea. Thicker lines denote data filtered by the 12-month moving average. The wind stress curl over the Weddell Sea is basically negative, and the barotropic streamfunction in the Weddell Sea is positive. The negative (positive) anomaly of wind stress curl indicates the enhanced (weakened) cyclonic circulation, but those of the barotropic streamfunction indicates spin-down (spin-up) of cyclonic Weddell Gyre, respectively. Blue triangles are indicative of the Maud Rise seamount.

We now address the question: why did the MRP occur only in 2017 and not during the above two earlier periods? As shown in Fig. 4a,b, the basin-scale cyclonic Weddell Gyre represents the horizontal circulation of the Weddell Sea. Although there is a difference of about 0.4 m between the SSHs of HYCOM and AVISO in the Weddell Sea, their overall patterns are similar, implying that the variation of the Weddell Gyre simulated by HYCOM is reliable. The southern Weddell Sea including the vicinity of Maud Rise is under the influence of the westward flowing limb. The atmosphere-to-ocean dynamic interaction triggered by the intensifying Southern Hemisphere westerly winds enhances the negative (cyclonic) wind stress curl over the Weddell Sea and the Weddell Gyre^31,34,35^. This plays a crucial role in upward doming of the pycnocline separating of the warm deep water from the colder surface layer, which is more conducive to the formation of WP. The wind stress curl anomaly is derived from the European Centre for Medium-Range Weather Forecast (ECMWF) ERA-Interim data^36^ and is averaged over the entire Weddell Sea (60°W–20°E/80°S–60°S; Fig. 4c). The wind stress curl anomaly does not vary much from 1995 to mid-2014 and then shows a dramatic decrease indicative of enhanced cyclonic circulation in 2015.

The Weddell Gyre presented as anomalous barotropic streamfunction remains constant from 1995 to mid-2014 and then intensifies, which is consistent with the overlying negative wind stress curl. In order to support this high-resolution reanalysis modeling results, the meridional geostrophic velocity is derived from the satellite-observed absolute dynamic topography and is averaged over 30°E–40°E/60°S–50°S, marking the eastern limb of the Weddell Gyre, an area unaffected by the seasonally varying sea ice (Fig. 4d). A negative anomaly denotes strengthening of the eastern limb of the Weddell Gyre, as the Weddell Gyre is intensified.

We conclude that the MRP2017 was generated by the wind-induced spin-up of the Weddell Gyre combined with weakly-stratified condition of the ocean near the Maud Rise. The weak stratification is a necessary condition for the occurrence of MRP, but not a sufficient condition. The MRP is formed when the Weddell Gyre is intensified by the atmosphere-to-ocean dynamic interaction and water column stratification is weakened.

### Maud Rise eddy activity

**Figure 5:**
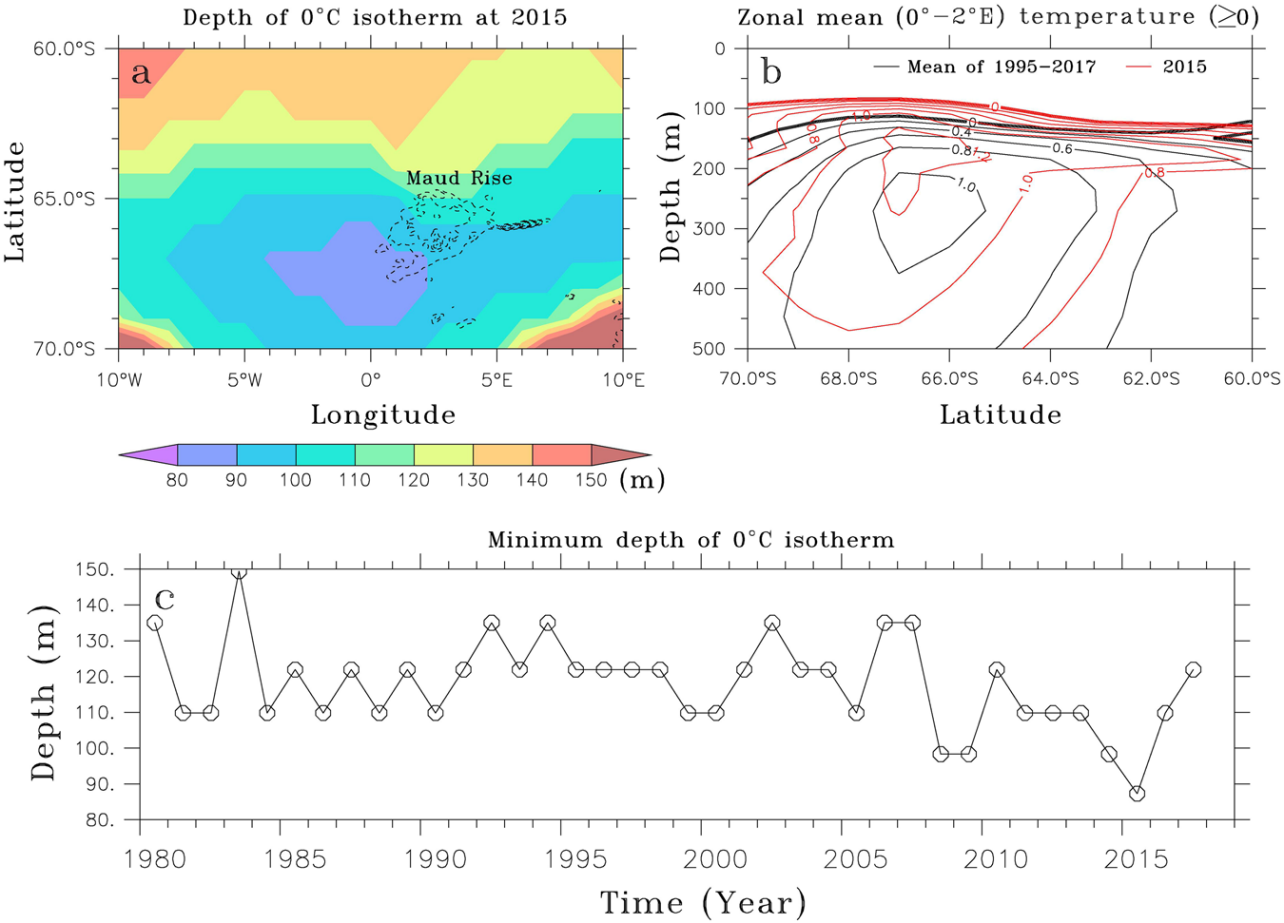
(**a**) The depth of the 0 °C isotherm in 2015, (**b**) zonal mean (0°–20°E), austral winter-mean (June/July/August) temperature (≥0) for (black) 1995–2017 and (red lines) 2015, and (**c**) time series of minimum depth of the 0 °C isotherm that is averaged over 0°–10°E/68°S–66°S. These are derived from the EN4.2.1 data.

Maud Rise plays an important role in enhancing upwelling of warm deep water via circulation/topography interaction over its flank^11,16,17,37–40^. The more vigorous Weddell Gyre previously shown can activate cyclonic eddies shed from the flank of Maud Rise^39^, which are expected to increase the Maud Rise-induced upwelling and to transmit divergent Ekman stress to the overlying sea ice cover^11^. The depth of the 0 °C isotherm calculated in the vicinity of Maud Rise for the 2015 winter (June/July/August) reveals shallowing of warm deep water in the southwestern flank of Maud Rise, indicative of a cyclonic eddy (Fig. 5a). The zonal mean (0°–2°E) temperature at 2015 is compared with that averaged from 1995 to 2017 in Fig. 5b. It shows the 0 °C isotherm shallowing by about 50 meters and the bottom of the 1 °C isotherm deepening by about 150 meters or more, indicating that the warm deep water layer is substantially thickened in 2015. A time series of the eddy activity estimated by the minimum depth of the 0 °C isotherm averaged over 0°E–10°E/68°S–66°S shows that it does not vary much until 2013 and then suddenly becomes most vigorous in 2015 (Fig. 5c). This is consistent with the Weddell Gyre strengthening induced by the negative wind stress curl, implying that the inflow to the Weddell Sea via its eastern limb plays a crucial role in activating the warm deep water eddy in the southwestern flank of Maud Rise, acting to enhance the probability of the MRP.

**Figure 6:**
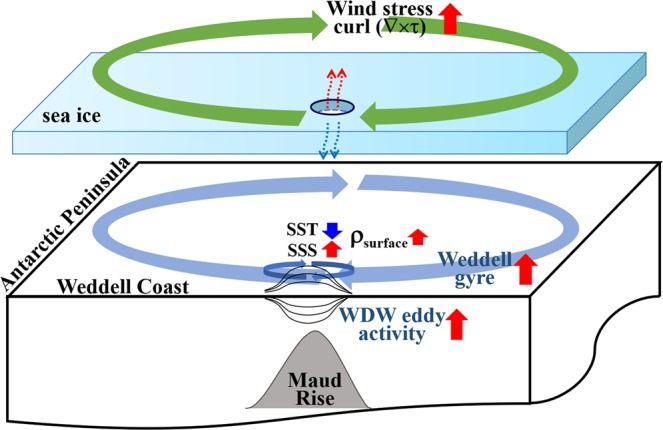
A simple schematic diagram to illustrate how an open-ocean polynya is triggered by both hydrological and dynamical processes.

As previously described in Fig. 2, the vertical profiles of temperature and salinity near the Maud Rise in April of 2016 provides precondition for MRP. Together with the increased surface water density, the Maud Rise cyclonic eddy in the austral winter of 2015 weakened the water column stratification, generating the precondition for the small-scale, late spring MRP in 2016 and 2017. In summary, since 1980 the ocean in the vicinity of Maud Rise became weakly-stratified over two periods: 2006 to 2009 and from 2013 to the present, in association with the surface cooling and salinization. However, only the weak stratification after 2013 was combined with the Weddell Gyre intensified by the overlying negative wind stress curl, weakening and shallowing the pycnocline, with intensification of warm deep water eddy near Maud Rise. The upwelled, warm WDW melts the overlying sea ice, leading to the MRP2017. The observational and reanalysis model data-based analysis illustrates the crucial roles that the increased SSS, the intensified Southern Hemisphere westerly winds, and the Maud Rise eddies play in the formation of open-ocean polynyas in the Weddell Sea. These processes are summarized in a schematic diagram (Fig. 6).

## Discussion

The life cycle of the MRP, from preconditioning to its eventual cessation, can be categorized by six stages as follows. (1) The upper ocean stratification weakens, providing the precondition for open-ocean polynya^29^. (2) The water column becomes sufficiently destabilized to trigger small-scale convection in the upper ocean, causing the relatively warm WDW below the pycnocline depth to rise to the surface^6^. (3) The upwelled warm deep water melts the overlying sea ice or prevents it from forming. As in the 1970s, the MRP serves as a precursor to the WP. (4) Once the MRP occurs, the relatively warm surface water (∼−1.8 °C) within the polynya is exposed to the cold winter atmosphere. (5) If there is insufficient introduction of freshwater by regional sea ice melt, oceanic deep convection ensues, and the corresponding ocean-to-atmosphere heat loss is enormous. Since the warmth of the WDW is essential for the maintenance of an open-ocean polynya, the drastic shortage of deep ocean heat content can be a crucial factor to eventually attenuate the open-ocean polynya. (6) In the final stage the open-ocean polynya disappears, and so does open-ocean deep convection, though the remnant impact of convection in the Weddell Sea persists until the WDW heat is restored by advection from the circumpolar ocean^12,35^.

**Figure 7:**
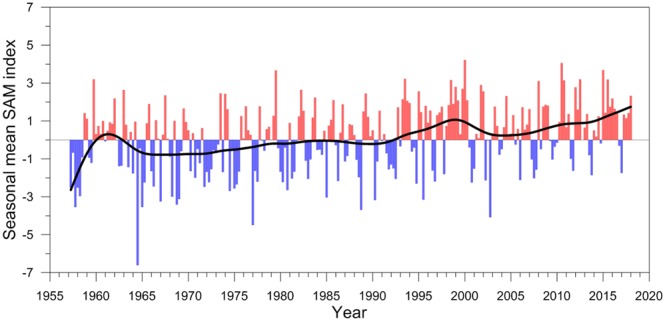
Time series of seasonal mean SAM index. The thick black line is calculated by the 10-year low pass filter.

Though conditions were right for the development of the MRP2017 event, the development of a subsequent WP in 2018 was hindered by the lack of larger scale regional conditioning. The surface salinity in the Weddell Sea correlates inversely with the SAM index^29^. The WP of the 1970s followed a prolonged, negative SAM, which induces a drier-than-normal atmosphere over the Weddell Sea, increasing the salinity of the cold surface layer, weakening the pycnocline separating it from the warmer deep water^27–29^. The weakened pycnocline acted to increase transfer of the ocean heat to the sea surface, adversely affecting the sea ice cover. As shown in Fig. 7, the SAM has long been in positive mode since the mid-1990s, and the SSS remained relatively fresh, hindering the MRP2017 ability to spark the larger-scale open-ocean WP. Moreover, the negative wind stress curl over the Weddell Sea reached its peak between 2015 and 2016 and started to weaken, and so did the Weddell Gyre. The activity of Maud Rise eddy reached its peak in 2015 and started to decline. If these had continued to intensify into 2018, the WP might have occurred. Finally, another factor (Fig. 2d) is that the deep water in the vicinity of Maud Rise already lost a huge amount of heat by April of 2018, indicating that the upwelled deep water may not have been warm enough to melt sea ice or to prevent it from forming during the 2018 austral winter season.

The previous study analyzing the historical observations and model simulations of the 5^th^ Coupled Model Intercomparison Project (CMIP5) expected open-ocean deep convection in the Southern Ocean to weaken and cease in association with the upper-ocean stratification proceeded by the surface freshening of the Antarctic ocean since the 1950s^19^. The surface freshening of the southern polar ocean may act to hinder the occurrence of open-ocean polynya in the future as well. However, according to the 5^th^ Assessment Report (AR5) of Intergovernmental Panel on Climate Change (IPCC), the SAM index has been gradually increasing since the late 1950s^41^, as also shown in Fig. 7. The increasing, positive SAM index is indicative of the Southern Hemisphere westerly winds that is shifted poleward and intensified, which implies an increasingly negative wind stress curl over the Weddell Sea. As illustrated in this paper, it activates the meso-scale WDW eddies in the vicinity of Maud Rise, which increases the possibility for the occurrence of an open-ocean polynya and thus conflicts with the prediction of aforementioned study^19^. We conclude that the combined effect of weakened thermohaline stratification with increased wind-induced Maud Rise eddy activity play a crucial role in generating open-ocean polynyas in the Southern Ocean. Therefore, between the surface freshening and the increasing, positive SAM index, both of which are predicted by CMIP5 models, which factor is a more crucial factor is an important question for predicting the occurrence of open-ocean polynyas.

## Methods

### Analyses of observation data

This study employed three observation data: (1) the HadISST data for sea ice concentration, (2) the EN4.2.1 data for temperature and salinity of the Weddell Sea, and (3) the AVISO data for geostrophic velocity derived from the absolute dynamic topography. These data downloaded from the respective websites were used in analysis without an additional preprocess.

Open-ocean polynya is defined as the ice-free area surrounded by sea ice, and its criterion is generally the area whose sea ice concentration is less than 20%. Both the 1970s’ WP and the MRP2017 satisfy this criterion. However, since the MRP is much smaller-scale open-ocean polynya and persists for much shorter period than the 1970s’ WP, its criterion is sometimes relaxed to be the pronounced occurrence of sea ice concentration below 92% in the vicinity of Maud Rise. According to this relaxed criterion, the MRPs can be stated to occur in 1980, 1991, 1994 and 2016 between the 1970s’ WP and the MRP2017, though their scale is even much smaller than the MRP2017. In order to focus on the MRP2017, the sea ice concentration from the HadISST is averaged over 0°–10°E/67°S–62°S where the MRPs usually occur and is used in Fig. 3a.

The EN4.2.1 dataset^30^ provided by the Met Office Hadley Centre consists of two products: (1) observed ocean temperature and salinity profiles and (2) objective analyses formed from the profile data with uncertainty estimates. Both products employ the bias adjustment correction for mechanical bathythermography (MBT) and expendable bathythermography (XBT) profiles. The Argo profile data of April of 2015/2016/2017/2018 is selected in the area where the MRP2017 occurred (3.5°–5.5°E/65.5°S–64°S) to show changes in the vertical distribution of temperature and salinity and in the ocean stratification (Fig. 2d,e) before (2015/2016/2017) and after (2018) the occurrence of MRP2107. The number of station data measured in April over the area is the most among the austral fall and winter seasons (from March to August), which is the reason for the April’s data to be selected. In order to assess the variation of surface ocean where the MRPs usually occur, the SST and SSS derived from the objective analyses form of EN4.2.1 are averaged over 0°–10°E/67°S–62°S, and their anomalies are calculated by subtracting the climatological mean of the analysis period (from January of 1980 to June of 2018) from the respective values and are shown in Fig. 3b with their 12-month moving averages. The density derived from the objective analyses form of EN4.2.1 are used in calculation of the potential energy anomaly^28^ (Eq. 1).

The meridional component of geostrophic velocities derived from gridded absolute dynamic topography (sea surface height above geoid) is employed in order to show the variation of the eastern limb of Weddell Gyre. Since a large portion of the Weddell Sea is covered by sea ice from the austral fall to spring, the sea surface height data observed by satellite cannot be used in assessing the variation of intensity of the Weddell Gyre. Therefore, the variation of the eastern limb of Weddell Gyre indicative of the gyre’s intensity is assessed by calculating the anomaly of the meridional geostrophic velocity averaged over 30°E–40°E/60°S–50°S (Fig. 4d), because the area is usually not covered by sea ice and is dominated by the southward flow of the gyre’s eastern limb. The northward flow of the gyre’s western limb is not available due to the eastern coast of Antarctic Peninsular entirely covered by sea ice from the austral fall to spring.

### Analyses of reanalysis data

In order to support the analysis of AVISO data that has a limitation of seasonally varying coverage, a high-resolution (1/12° × 1/12° in horizontal direction) reanalysis data provided by the Naval Research Laboratory are employed to calculate the horizontal barotropic streamfunction indicative of intensity of the Weddell gyre. The reanalysis system is configured for the global ocean with HYCOM 2.2 as the dynamical ocean model. The HYCOM has 32 vertical layers, and its bathymetry is derived from the General Bathymetric Chart of the Ocean (GEBCO) dataset. The surface forcing including wind stress, wind speed, heat flux, and precipitation is derived from one-hourly National Centers for Environmental Prediction (NCEP) Climate Forecast System Reanalysis (CFSR). This system uses the Navy Coupled Ocean Data Assimilation (NCODA) system for data assimilation^42,43^. The maximum value of barotropic streamfunction over 60°W–20°E/80°S–60°S is used for the intensity of Weddell Gyre (Fig. 4c).

## Data Availability

The sea ice concentration of HadISST data is available at the Met Office Hadley Centre (https://www.metoffice.gov.uk/hadobs/hadisst/). The profile and objective analyses forms of the EN4.2.1 is also provided by the Met Office Hadley Centre and is available at https://www.metoffice.gov.uk/hadobs/en4/. The monthly mean data sets of ERA-Interim are provided by ECMWF and are available at http://apps.ecmwf.int/datasets/data/interim-full-moda/levtype=sfc/. The 1/12 deg global HYCOM + NCODA Ocean Reanalysis data is available at https://hycom.org/dataserver/gofs-3pt0/analysis. The observation-based SAM index data is available at http://www.nerc-bas.ac.uk/icd/gjma/sam.html.
